# Inhibition Analysis and High-Resolution Crystal Structure of *Mus musculus* Glutathione Transferase P1-1

**DOI:** 10.3390/biom13040613

**Published:** 2023-03-29

**Authors:** Oleksii Kupreienko, Fotini Pouliou, Konstantinos Konstandinidis, Irene Axarli, Eleni Douni, Anastassios C. Papageorgiou, Nikolaos E. Labrou

**Affiliations:** 1 Laboratory of Enzyme Technology, Department of Biotechnology, School of Applied Biology and Biotechnology, Agricultural University of Athens, 75 Iera Odos Street, 11855 Athens, Greece; 2 Laboratory of Genetics, Department of Biotechnology, School of Applied Biology and Biotechnology, Agricultural University of Athens, 75 Iera Odos Street, 11855 Athens, Greece; 3 Alexander Fleming, Institute for Bioinnovation, Biomedical Sciences Research Center, 16672 Vari, Greece; 4 Turku Bioscience Centre, University of Turku and Åbo Akademi University, 20521 Turku, Finland

**Keywords:** anticancer drugs, cancer, human glutathione transferase P1-1 (hGSTP1-1), glutathione transferase, enzyme inhibition, multidrug resistance, pesticide

## Abstract

Multidrug resistance is a significant barrier that makes anticancer therapies less effective. Glutathione transferases (GSTs) are involved in multidrug resistance mechanisms and play a significant part in the metabolism of alkylating anticancer drugs. The purpose of this study was to screen and select a lead compound with high inhibitory potency against the isoenzyme GSTP1-1 from *Mus musculus* (*Mm*GSTP1-1). The lead compound was selected following the screening of a library of currently approved and registered pesticides that belong to different chemical classes. The results showed that the fungicide iprodione [3-(3,5-dichlorophenyl)-2,4-dioxo-N-propan-2-ylimidazolidine-1-carboxamide] exhibited the highest inhibition potency (ΙC_50_ = 11.3 ± 0.5 μΜ) towards *Mm*GSTP1-1. Kinetics analysis revealed that iprodione functions as a mixed-type inhibitor towards glutathione (GSH) and non-competitive inhibitor towards 1-chloro-2,4-dinitrobenzene (CDNB). X-ray crystallography was used to determine the crystal structure of *Mm*GSTP1-1 at 1.28 Å resolution as a complex with S-(p-nitrobenzyl)glutathione (Nb-GSH). The crystal structure was used to map the ligand-binding site of *Mm*GSTP1-1 and to provide structural data of the interaction of the enzyme with iprodione using molecular docking. The results of this study shed light on the inhibition mechanism of *Mm*GSTP1-1 and provide a new compound as a potential lead structure for future drug/inhibitor development.

## 1. Introduction

A large superfamily of enzymes known as glutathione transferases (GSTs; EC 2.5.1.18) catalyze the nucleophilic attack of the reduced tripeptide glutathione (GSH, L-Glu-L-Cys-Gly) on nonpolar molecules that contain an electrophilic carbon, oxygen, or sulfur atom [1,2,3]. GSTs play a crucial role in Phase II of the cell detoxification process and are considered essential components of this process [4,5,6]. Beyond their primary detoxification function, some GSTs are also involved in the biosynthesis of hormones in mammals, catabolism of amino acids, signalling pathways, synthesis of prostaglandins and leukotrienes, breakdown of reactive oxygen species (ROS), and other significant intracellular processes [3].

GSTs are primarily classified based on their structure and sequence [1]. They are classified into three separate families, cytosolic, mitochondrial, and microsomal GSTs, with members found in various kingdoms and phyla. The cytosolic family is comprised of the following classes: α (alpha), β (beta), δ (delta), ε (epsilon), ζ (zeta), θ (theta), μ (mu), ν (nu), π (pi), σ (sigma), τ (tau), φ (phi), and ω (omega) [1,3,5].

GSTs function as dimers consisting of two similar or different subunits, each with an average sequence length of 200–250 amino acids. Each subunit of GSTs has at least two binding sites, the G-site and the H-site [1,5,7], which are able to bind GSH and the electrophilic substrate, respectively. The G-site is located at the N-terminal region and its structure is strictly conserved among GSTs that belong to different classes. It consists of β-strands (β1, β2, β3, and β4), three of which are antiparallel to each other (β1, β2, and β3). This structural region of the β-strands is sandwiched between α-helices, resulting in a βαβαββα structural motif. The C-terminal region is entirely helical and is formed by five or six α-helices. Some GST classes, such as alpha, omega, tau, and theta may have an additional α-helix. The H-site is located at the C-terminal region. Its structure is less conserved than that of the G-site and contributes to the broad specificity of GSTs towards electrophilic substrates [1,8,9,10]. 

The catalytic role of GSTs in multidrug resistance (MDR) of cancer cells is mainly due to their overexpression, leading to rapid detoxification of anticancer drugs [9,11,12]. The GST classes that are associated with the development of MDR cancer cells in humans are alpha, mu, and pi [13,14,15]. Human GSTP1-1 (hGSTP1-1) is the most well-studied member of the GST family. It is involved in apoptosis resistance and metabolism of various chemotherapeutic agents, such as platinum-based drugs [16,17]. In addition, hGSTP1-1 regulates calcium channels by reducing the apoptotic mobilization of calcium ions [18] and modulating the function of apoptotic signalling of JNK1 [19,20] and Bax [21]. Additionally, hGSTP1-1 regulates calcium channels by reducing the apoptotic mobilization of calcium ions [18], modulating the function of apoptotic signalling of JNK1 [19,20] and Bax [21]. The regulation of tumor necrosis factor (TNF), TNF-receptor factor 2 (TRAF2), and apoptosis signal-regulating kinase 1 (ASK1) is another crucial function of hGSTP1-1 [22]. Through a redox mechanism, nuclear factor (NF)-κB and activator protein 1 both mediate the control of hGSTP1-1 [23]. Therefore, identifying GSTP1-1 inhibitors may be valuable for developing new therapeutic strategies for cancer [5,17,20]. 

A range of synthetic and natural substances has been tested for their ability to inhibit GSTs in an attempt to lessen or even abolish multidrug resistance in cancer cells. 2,2′-dihydroxybenzophenones [24], benzoxazole [25], selenium compounds [26], diselenides and benzisoselenazolones [27], benzoxadiazoles [28,29], auranofin [30], piperlongumine [31], and curcumin analogs [32,33] have been recently reported.

The use of approved compounds (e.g., FDA-approved drugs, pesticides) as a source of molecular scaffolds is an effective way to reduce the cost and time required for new lead development. Reuse of existing chemical scaffolds, with chemical and toxicological profiles that are already established, can provide a significant advantage in terms of safety and reduce the risk associated with new drug development. For example, Musdal et al. [34], in a library of FDA-approved drugs, have identified merbromine, hexachlorophene, and ethacrynic acid as the most effective hGSTP1-1 inhibitors with IC_50_ values in the low micromolar range. Recently, Bodourian et al. [33], using the human GSTM1-1 (hGSTM1-1) as a model enzyme, exploited a diverse pesticide library to identify the carbamate insecticide pirimicarb as a strong inhibitor of hGSTM1-1. The present study aimed to identify a new lead compound from a pesticide library as an inhibitor of *Mus musculus* GSTP1-1 (*Mm*GSTP1-1) and provide the basis for further research and development.

## 2. Materials and Methods

### 2.1. Materials

Glutathione (reduced form, GSH), 1-chloro-2,4-dinitrobenzene (CDNB), acetochlor, butachlor, metazachlor, and bovine serum albumin (BSA, fraction V) were purchased from Sigma-Aldrich (St. Louis, MI, USA). Alachlor and malathion were purchased from Fluka (Darmstadt, Germany). Other pesticides were obtained from Riedel de Haen (Hanover, Germany).

### 2.2. Methods

#### 2.2.1. Cloning, Expression, and Purification of MmGSTP1-1

First-strand cDNA synthesis was performed using Poly(A)-mRNA, oligo-p(dT)15 primer and Superscript II reverse transcriptase according to the manufacturer’s recommendations (Thermo Fisher Scientific, Waltham, MA, USA). The PCR-primers: 5′ GAA GGA GAT ATA CAT ATG ATG GCC GGG AAG CCC GTG CTT CAC-3′ (forward primer) and 5′-GTG ATG GTG GTG ATG ATG ΤΤA TCA CTG AAT CTT GAA AGC CTT CCT TGC TTC-3′ (reverse primer) were designed according to the *Mm*GSTP1-1 gene sequence (GenBank accession number: BC061109.1, accessed on 5 October 2016). The PCR reaction was carried out in a total volume of 50 μL containing 10 pmol of each primer, 1μg template cDNA, 0.2 mM of each dNTP, 5 μL 10× buffer, and 1 unit of Accura High-Fidelity DNA polymerase (Lucigen, Middlesex, UK). The PCR procedure comprised of 25 cycles of 45 s at 94 °C, 15 s at 65 °C, and 1 min at 72 °C. A final extension time at 72 °C for 10 min was performed after the 25 cycles. The PCR products were run on a 1% (*w*/*v*) agarose gel, and the single PCR product (630 bp) was excised, purified by adsorption to silica beads and cloned to the expression plasmid pETite C-His. The resulting expression construct was used to transform competent *E. coli* HI-Control BL21 (DE3) cells for expression. Nucleotide sequencing was performed along both strands for sequence validation.

Competent *E. coli* HI-Control BL21 (DE3) cells were grown in Luria–Bertani (LB) medium containing kanamycin (25 μg/mL). GST synthesis was induced by the addition of 1 mM isopropyl-β-D-thiogalactopyranoside (IPTG) when the absorbance at 600 nm had reached 0.6. Following incubation, the cells were harvested by centrifugation at 8000× *g* for 20 min, resuspended in phosphate buffer (20 mM, pH 7), sonicated, and centrifuged at 13,000× *g* for 5 min. The supernatant was loaded onto an epoxy-activated Sepharose CL-6B column coupled to GSH (1,4-butanediol diglycidyl ether-GSH-Sepharose-CL6B, 1 mL), which was previously equilibrated with potassium phosphate buffer (20 mM, pH 7). The bound enzyme was eluted by the equilibration buffer containing GSH (1 mM). The purity of the protein was judged by SDS-PAGE (Supplementary Figure S1).

#### 2.2.2. Assay of Protein and Enzyme Activity

Determination of enzyme activity was performed by monitoring the formation of a conjugate between GSH (2.5 mM) and CDNB (1 mM) at 340 nm (ε = 9.6 mM^−1^cm^−1^) at 37 °C, pH = 6.5, using a published method [35]. Observed reaction velocities were corrected for spontaneous reaction rates when necessary. Turnover numbers were calculated based on the presence of one active site per subunit. All the initial velocities were determined at least three times in buffers equilibrated at a constant temperature. As defined, one enzyme unit is the amount of enzyme that produces 1 μmole of product per minute at pH = 6.5 and 37 °C.

#### 2.2.3. Pesticides Screening

In total, 49 pesticides were included in the analysis. Inhibition potency evaluation was carried out in the same assay system as described above in the presence of 25 μM of pesticide dissolved in acetone. The percentage of enzyme inhibition was calculated as follows:

%(Inhibition) = [(R_u_ − R_i_)/R_u_] × 100, (1)

where R_u_ is the rate of absorbance increase for the reaction in the absence of the inhibitor and R_i_ is the rate of absorbance increase for the reaction in the presence of the inhibitor.

Both R_u_ and R_i_ were measured at the same substrate concentration (GSH and CDNB). No reaction between GSH and pesticide was observed during the enzyme assay (60 s).

#### 2.2.4. IC_50_ Value Determination for Iprodione

The IC_50_ value of iprodione was determined using the assay system described above [35], and the *Mm*GSTP1-1 activity was measured at different concentrations of iprodione (0–75 μM). The IC_50_ value was calculated by fitting the following equation to the concentration-response data:

%(Inhibition) = 100/[1 + (IC_50_/[I])], (2)

where [I] is the concentration of the inhibitor (e.g., iprodione). The IC_50_ value was determined by fitting the above equation to the experimental data using a non-linear curve fitting method via GraphPad Prism v7.00 (GraphPad Software Inc., Boston, MA, USA).

#### 2.2.5. Kinetic Inhibition Study of MmGSTP1 in Presence of Iprodione

A kinetic study of *Mm*GSTP1-1 using GSH as a variable substrate was performed using the assay system mentioned above with different GSH concentrations (18.75–3375 μM) in the presence of different (0–10 μM) concentrations of iprodione. Using CDNB as a variable substrate, the same assay system was used with different CDNB concentrations (30–1500 μM) in the presence of variable (0–10 μM) concentrations of iprodione. The kinetic parameters (K_m_, k_cat_) were determined via GraphPad Prism v7.00 (GraphPad Software Inc.)

#### 2.2.6. X-ray Crystallography 

*Mm*GSTP1-1 was co-crystallized in the presence of 2.5 mM Nb-GSH using the hanging drop vapor diffusion method. The well solution consisted of 0.1 M Tris-HCl, PEG 6000 20% (*w*/*v*) and 0.2 M calcium chloride dihydrate, pH 8.0. X-ray diffraction data from a single crystal were collected on the P13 beamline (EMBL-Hamburg) to 1.28 Å resolution at 100 K using glycerol 10% (*v*/*v*) as cryoprotectant. The initial phases were determined using molecular replacement with the previously determined structure of mouse liver GSTP1-1 at 1.8 Å resolution (PDB ID: 1GLQ; sequence identity 100%) as a search model in Phaser [36]. The crystals of the 1GLQ structure also belong to the orthorhombic *P*2_1_2_1_2_1_ space group with similar unit cell dimensions as the ones reported here but the axes have been assigned differently. The structure was refined with Phenix v. 1.20.1-4487 [37].

#### 2.2.7. Molecular Docking of Iprodione

Molecular docking of iprodione was performed using AutoDock4.2 (version 4.2.6) and AutoDockTools4 [38]. Ligand-free *Mm*GSTP1-1 and iprodione were used as the receptor and ligand, respectively. The grid was centred at −31, 0, 23 coordinates (with grid sides having 100, 125, and 100 points, spaced at 0.375 Å). AutoDock4.2 was then used for the docking analysis, carrying 100 independent genetic algorithm cycles with a population of 300 individuals. The docked ligand clusters were then further analyzed using PyMOL [39]. The refined crystal structure of *Mm*GSTP1-1 resolved in the present study at 1.28 Å resolution was used. 

## 3. Results and Discussion

### 3.1. Purification and Kinetic Analysis 

**Figure 1:**
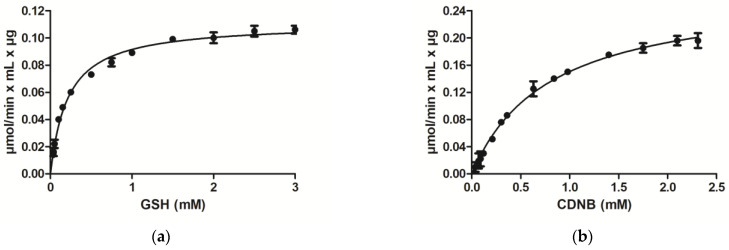
(**a**) Kinetics analysis of *Mm*GSTP1-1 using CDNB at saturated concentration and GSH varied. (**b**) Kinetics analysis of *Mm*GSTP1-1 using GSH at saturated concentration and CDNB varied.

Recombinant *Mm*GSTP1-1 was purified in a single step by affinity chromatography using Sepharose-immobilized GSH as an affinity ligand. Kinetics analysis was carried out using the model GST compounds CDNB and GSH as substrates. The enzyme was found to obey Michaelis–Menten kinetics (Figure 1) with K_m_^CDNB^ 0.80 ± 0.04 mM and K_m_^GSH^ 0.22 ± 0.01 mM. Both parameters are close to those reported for the hGSTP1-1 isoenzyme [40,41,42]. 

The substrate specificity of the purified enzyme was assessed using a panel of model GST substrates. The results are presented in Table 1. *Mm*GSTP1-1 exhibits a range of diverse activities including transferase activity towards alkyl-halides or unsaturated compounds and hydroperoxidase activity using cumene hydroperoxide as substrate. GSTs are known to participate in oxidative stress defense mechanisms, catalyzing the GSH-dependent inactivation of organic hydroperoxides, such as cumene hydroperoxide and converting them into non-toxic alcohols [43]. tert-Butyl-hydroperoxide did not appear to be an acceptable substrate for the enzyme. Significant activity is exhibited towards ethacrynic acid, a well-known inhibitor of GSTs [34]. Significant activity was also observed with phenethyl isothiocyanate as a substrate, suggesting that *Mm*GSTP1-1 catalyzes with high efficiency the addition of the thiol of GSH to the electrophilic central carbon of the isothiocyanate group. Similar substrate specificity towards the reported electrophilic substrates has also been observed for hGSTP1-1 [40,41,42,44].

### 3.2. Pesticides Library Screening

**Figure 2:**
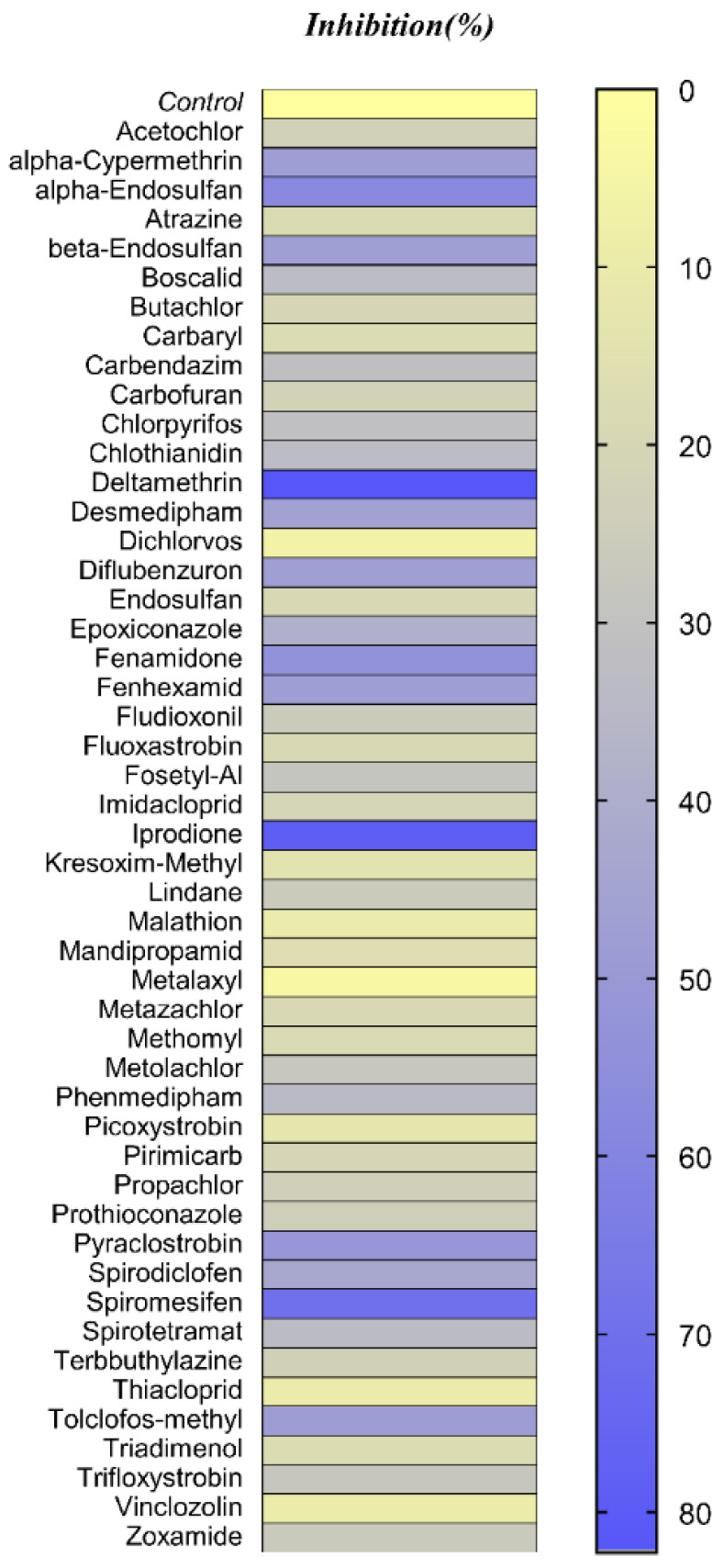
Screening of the inhibitory potency of pesticides (25 μM) against *Mm*GSTP1-1. The enzyme was assayed in all measurements using the CDNB-GSH assay system. The colour scale represents the mean values of three inhibition assays (%) for each pesticide against the tested enzyme with a variation of less than 5% in all cases.

A library of 49 different pesticides belonging to diverse chemical classes, including insecticides, herbicides, and fungicides, was selected to assess their inhibitory potency against the *Mm*GSTP1-1 isoenzyme. The selected pesticides represent a mixed collection of compounds with diverse structural and physicochemical properties. The results of the screening are illustrated in Figure 2 in the form of a heatmap.

The results showed that the insecticide deltamethrin and the fungicide iprodione display the highest inhibition potencies, 82% and 78%, respectively. Considering that among deltamethrin and iprodione, the latter displays much less toxicity [e.g., acute oral toxicity LD_50_ (rat) > 5 g/kg and acute dermal toxicity LD_50_ (rabbit) > 2 g/kg], iprodione was selected for further studies. 

Other pesticides with significant but much lower inhibition potency compared to iprodione, include spiromesifen (70% inhibition), α-endosulfan (58% inhibition), and fenamidone (53% inhibition). 

**Figure 3:**
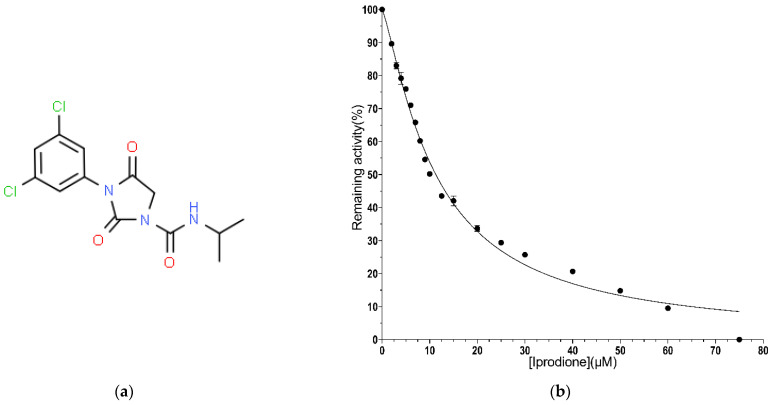
(**a**) The 2D structure of iprodione (C_13_H_13_Cl_2_N_3_O_3_). (**b**) Concentration–response curve for iprodione. The IC_50_ for *Mm*GSTP1−1 was determined by fitting Equation (2) to the data using nonlinear curve-fitting methods.

The concentration–inhibition curve for iprodione allowed the determination of the half maximal inhibitory concentration (IC_50_) which was 11.3 ± 0.5 μM (R^2^ = 0.989) (Figure 3). This value lies within the range expected for a strong GST inhibitor [24,25,27,32,33].

### 3.3. Kinetic Inhibition Study

**Figure 4:**
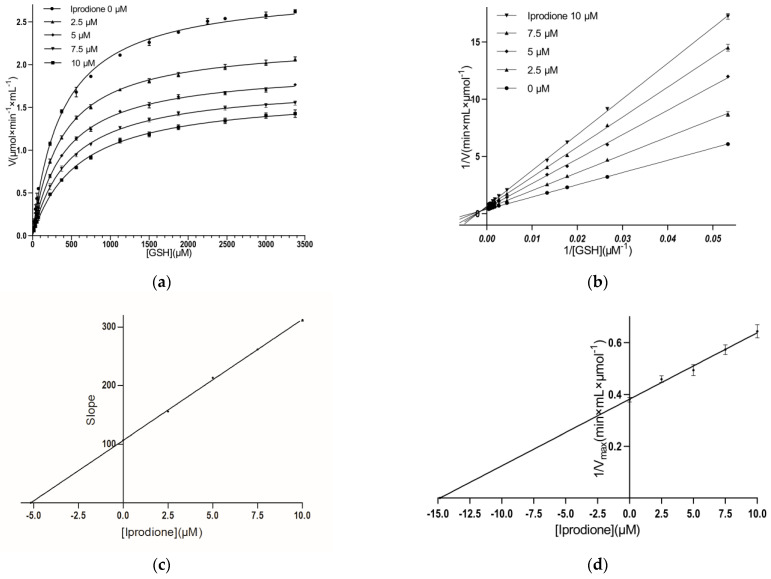
Kinetic inhibition of *Mm*GSTP1−1 by iprodione. The concentration of CDNB was constant, while the concentration of GSH was varied. (**a**) Michaelis–Menten plot and (**b**) Lineweaver–Burk plot for the inhibition of *Mm*GSTP1-1 with iprodione at different GSH concentrations. Iprodione concentrations: 0–10 μΜ. (**c**) Secondary plot for the determination of K_iE_ for the GSH. The slope values were obtained from a Lineweaver–Burk plot. (**d**) Secondary plot for the determination of K_iES_ for the GSH. The 1/V_max_ values are the intersection points of lines on the ordinate axis of the Lineweaver–Burk plot.

**Figure 5:**
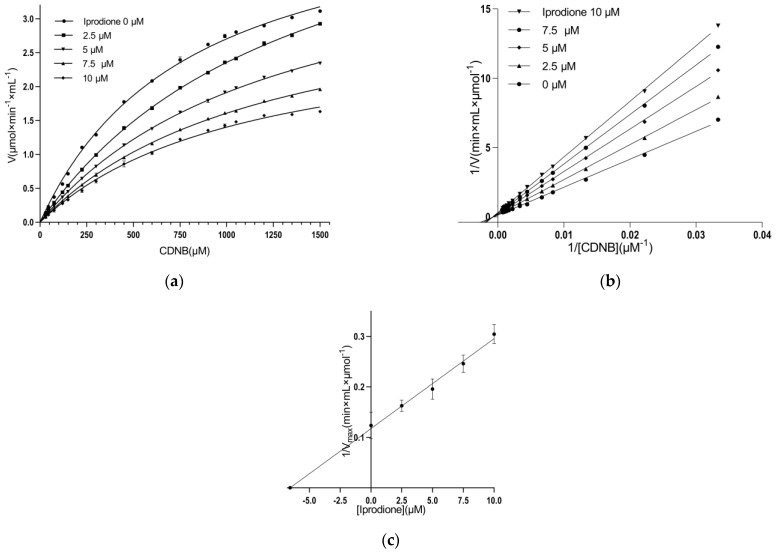
Kinetic inhibition study of *Mm*GSTP1−1 with iprodione. The concentration of GSH was constant, while the concentration of CDNB was varied. (**a**) Michaelis–Menten plot. (**b**) Lineweaver–Burk plot for inhibition. Iprodione concentrations: 0–10 μΜ. (**c**) Secondary plot for K_i_ determination.

A kinetic inhibition study was performed with the aim of determining the inhibition pattern of iprodione with the enzyme, and the results are illustrated in Figure 4 and Figure 5. The data suggest that iprodione behaves as a mixed-type inhibitor towards GSH (Figure 4) and a non-competitive inhibitor towards CDNB (Figure 5).

The mixed-type inhibitor indicates that iprodione can bind to both the free enzyme and the enzyme–substrate complex (E-GSH) with different affinities for each enzyme (K_iE_ ≠ K_iES_). The analysis reveals that the inhibition constants K_iE_ and K_iEGSH_ for iprodione, using GSH as a variable substrate, are 5.2 ± 0.1 μM (R^2^ = 0.99) and 14.8 ± 4 μM (R^2^ = 0.99), respectively. 

The measured K_i_ values (K_iE_ and K_iEGSH_) suggest that iprodione shows preferential binding and greater affinity for the free form of *Mm*GSTP1-1. This suggests that the recognition elements of binding for the inhibitor are not completely overlapping with those of the substrate, and therefore, the substrate and inhibitor binding sites may be thought to be distinct. Thus, the binding of iprodione to *Mm*GSTP1-1 may not preclude GSH binding. The non-competitive inhibition constant using CDNB as a variable substrate is K_i_ = 6.6 ± 2.6 μM (R^2^ = 0.98). 

It is well established that the inhibition pattern that is obeyed by different GST isoenzymes when studied with different inhibitory compounds is inhibitor- and substrate-dependent and cannot be predicted based on structural information of the particular inhibitor or the enzyme. However, the vast majority of kinetic inhibition studies have revealed that the most potent inhibitors function as non-competitive/mixed-type inhibitors [24,25,27,32,33]. 

### 3.4. Crystallization of MmGSTP1-1 and Structural Analysis 

The crystal structure of *Mm*GSTP1-1 was refined to 1.28 Å resolution with final *R*_work_ and *R*_free_ values of 0.175 and 0.199, respectively (Table 2). *Mm*GSTP1-1 was crystallized with two molecules in the asymmetric unit. The final refined structure contains 4083 non-hydrogen atoms. Of them, 3338 belong to the protein and 602 are water molecules. Compared to the previously determined *Mm*GSTP1-1 structure (PDB ID: 1GLQ, 1.8 Å resolution) [45] which has 148 water molecules, the high-resolution structure reported here shows a significant improvement in the number of water molecules. 

**Figure 6:**
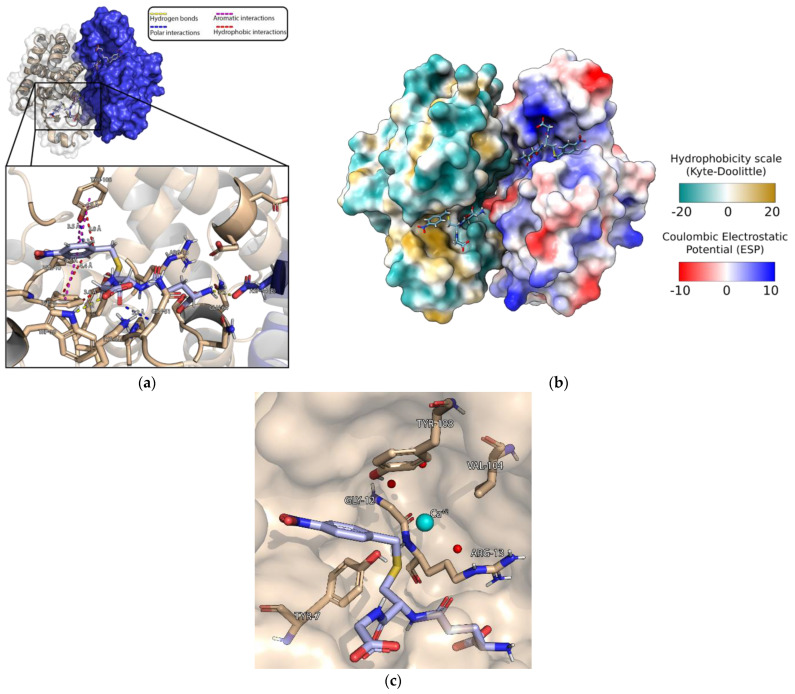
(**a**) The structure of *Mm*GSTP1−1 at 1.28 Å resolution. Each subunit is shown in a different color. The bound Nb-GSH is shown in a stick representation and colored according to the atom type. The figure was produced using the PyMOL program [39]. Close up: The amino acids that contribute to Nb-GSH binding. The close-up figure was created using PyMOL [39]. Interactions were calculated using the web server Arpeggio [46]. (**b**) The electrostatic and hydrophobicity potential of *Mm*GSTP1-1. One subunit is colored according to the electrostatic potential. Negative, positive, and neutral charges are shown as shades of red, blue, and white, respectively. The other subunit is colored according to the hydropathic character of the protein [47]. Hydrophilic to hydrophobic residues are colored from pale green to yellow brown. The bound NB-GSH is shown in a stick representation. The figure was created using the UCSF ChimeraX program [48,49]. (**c**) The interactions of the Ca^2+^ in the vicinity of H-site with important amino acid residues. Ca^2+^ is shown as a green sphere and water molecules as red spheres. Nb-GSH is shown in a stick representation and colored according to the atom type.

A molecule of Nb-GSH was found bound in the active site of each molecule of the dimer (Figure 6a,b; Supplementary Figure S2). A structural comparison with the previously determined *Mm*GSTP1-1 structure at a lower resolution (1.8 Å) [45] showed a root mean square deviation (RMSD) between the two structures of 0.315 Å for 209 atom pairs, suggesting subtle differences between them.

The amino acid residues that contribute to the binding of Nb-GSH are strictly conserved in all mammalian GSTP1-1 enzymes as well as in *Mm*GSTP1-1 (Figure 6a). Notably, Tyr108 makes a π-π aromatic–aromatic interaction with the nitrobenzyl group of the bound Nb-GSH. 

An interesting finding that has not been described previously is the presence of four calcium ions in the present structure. The source of the calcium ions is the salt used during crystallization. Among them, one calcium ion was found in each of the active sites (Figure 6c). Their B-factors were 21.4 and 28.2 Å^2^, indicating stable binding. Each of them is located in the vicinity of the H-site and forms 24 van der Waals interactions in total with protein atoms. Important van der Waals interactions are formed with the hydroxyl groups of the key residues Tyr7 and Tyr108 and with the side chains of Val104 and Arg13. In addition, the calcium ion interacts with the sulfur group of Nb-GSH. 

All these interactions seem to contribute towards stabilizing and fixing the orientation of the aromatic groups of Tyr7 and Tyr108, allowing the formation of aromatic–aromatic interactions with the nitrobenzyl group of the bound Nb-GSH. 

### 3.5. Characterization of the Iprodione Binding Site Using Molecular Docking

The crystal structure of *Mm*GSTP1-1 that was determined in the present study was used to map the iprodione-binding site using molecular docking. As co-crystallization of the *Mm*GSTP1-1/iprodione complex was not achieved despite extensive crystallization screens, an in silico molecular docking approach was adopted using AutoDock 4.2 [38].

**Figure 7:**
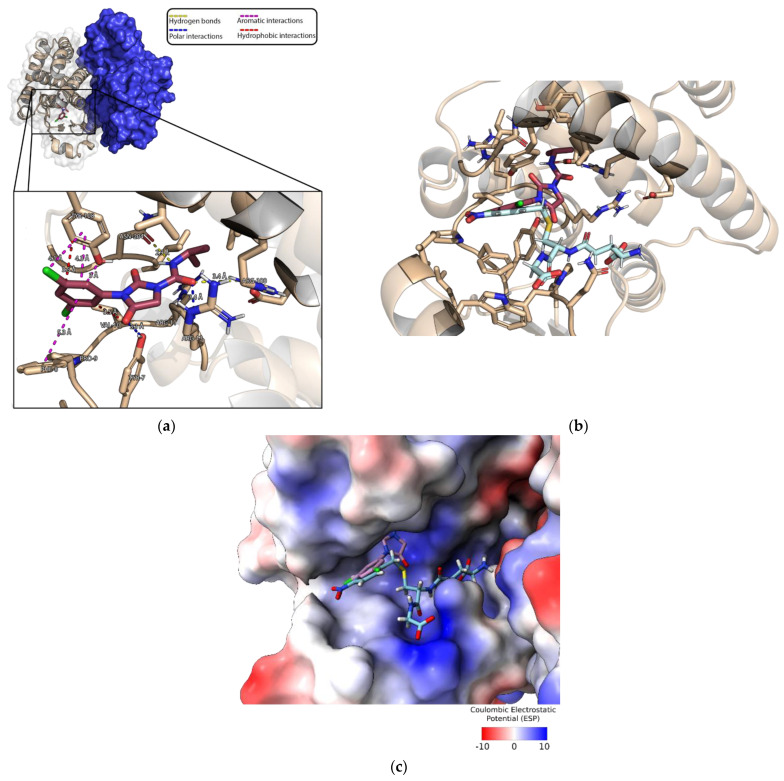
(**a**) Predicted favorable binding mode of iprodione in *Mm*GSTP1−1. Iprodione is shown in a stick representation. Close up: Side chains of the interacting amino acids are shown and labelled. Interactions were calculated using the Arpeggio web server [46]. The dashed lines represent interactions. (**b**) Superposition of bound iprodione and Nb-GSH in *Mm*GSTP1-1. Both ligands are shown in stick representation. Side chains of the interacting amino acids are shown. (**c**) Superposition of bound iprodione and Nb-GSH. Both ligands are shown in stick representation. The enzyme subunit is shown as the surface and colored according to the Coulombic electrostatic potential. Negative, positive, and neutral charges are shown as shades of red, blue, and white, respectively.

Based on the most credible conformation cluster, iprodione appears to bind to the region that partially overlaps with the H-site (Figure 7a). Its aromatic ring is sandwiched between the aromatic groups of Tyr108 and Phe8. It also forms two hydrogen bonds with residues Arg100 and Asn204 and two polar interactions with residues Tyr7 and Arg13 (Figure 7a). Superposition of the binding site of iprodione and Nb-GSH showed that iprodione overlaps only with the nitrobenzyl group of the bound Nb-GSH (Figure 7b,c).

This binding mode is consistent with the observed mixed-type inhibition towards GSH and non-competitive inhibition towards CDNB, as found in the present study (Figure 5).

Based on the estimation made by AutoDock 4.2, the free energy of binding is equal to −7.17 kcal/mol and the inhibition constant K_i_ is equal to 5.68 μM. This theoretical value is very close to that which was measured experimentally for iprodione using GSH as a variable substrate (K_iE_ = 5.2 ± 0.1 μM) and using CDNB as a variable substrate (K_i_ = 6.6 ± 2.6 μM), supporting the accuracy of the in silico docking studies.

## 4. Conclusions

The discovery of GST inhibitors remains a challenging task [50]. This is probably a consequence of the high plasticity and conformational flexibility of GSTs. The results of the present study allowed the identification of the fungicide iprodione as an inhibitor of *Mm*GSTP1-1.

Iprodione acts as a mixed-type inhibitor towards GSH and as a non-competitive inhibitor towards CDNB. X-ray crystallography was used to determine the crystal structure of *Mm*GSTP1-1 in complex with Nb-GSH and molecular docking was used to investigate the interaction between *Mm*GSTP1-1 and iprodione.

Iprodione exhibits minimal toxicity to mammals and, therefore, can be considered as a new lead compound that can be further investigated. Repurposing already approved drugs seems to be a very attractive approach given the investment (cost, time, and effort) required for implementing a new drug discovery project. In this way, the drug development timeline can be shortened since several existing compounds already have known technology, safety, and toxicity profiles.

## Figures and Tables

**Table 1:** Substrate specificity of *Mm*GSTP1-1. The results represent the mean of triplicate determinations, with a variation of less than 5% in all cases. The specific activity of the enzyme against the model substrate CDNB is defined as 100%.

Electrophile Substrates	U/mg (%)
1-Chloro-2,4-dinitrobenzene	100
1-Bromo-2,4-dinitrobenzene	188.8
1-Iodo-2,4-dinitrobenzene	14.4
4-Chloro-7-nitrobenzofurazan	-
p-Nitrobenzyl chloride	1.7
Bromosulfalein	8.7
Cumene hydroperoxide	2.7
tert-Butyl hydroperoxide	-
Dehydroascorbate	-
Sulphanilamide	0.2
2,3-Dichloro-4-[2-methylene-butyryl]phenoxy)acetic acid (Ethacrynic acid)	19.1
*trans*-4-Phenyl-3-buten-2-one	-
Allyl isothiocyanate	-
Phenethyl isothiocyanate	41.1

**Table 2:** X-ray data collection and refinement statistics.

Data Collection	*Mm*GSTP1-1
Beamline	P13 (EMBL, Hamburg)
Wavelength (Å)	1.033
Resolution (Å)	56.62–1.28 (1.30–1.28)
Space group	*P*2_1_2_1_2_1_
Unit cell (Å) a, b, c	56.62, 77.37, 101.44
No. of unique reflections	114,851 (5532)
Completeness (%)	99.7 (98.3)
Multiplicity	6.4 (6.3)
Mosaicity (°)	0.11
*R*_meas_	0.059 (2.485)
CC_1/2_	0.99 (0.35)
Mean (I/σ(I))	12.7 (0.9)
Wilson B-factor (Å^2^)	18.3
**Refinement**	
No. of reflections used	114,744
*R*_work_/*R*_free_	0.175/0.199
No. of non-H atoms (protein/ligand/solvent)	3338/143/602
Protein residues	418
RMSD in bonds (Å)	0.006
RMSD in angles (°)	0.93
Average B-factor (all/protein/ligands/solvent) (Å^2^)	28.2/26.6/28.8/36.8
Ramachandran favored/outliers (%)	97.8/0.0
Rotamer outliers (%)	0.0
Clashscore	3.5
PDB ID	8C5D

## Data Availability

Data is contained within the article or Supplementary Materials.

## Supplementary Materials

The following supporting information can be downloaded at: https://www.mdpi.com/article/10.3390/biom13040613/s1, Figure S1. SDS-PAGE analysis of the eluted fraction of recombinant MmGSTP1-1; Figure S2. SigmaA-weighted 2Fo-Fc electron density map of Nb-GSH with surrounding residues at 1 sigma level. The figure was created using Coot [51].

## Abbreviation

CDNB, 1-chloro-2, 4-dinitrobenzene; GSH, glutathione; GST, glutathione transferase; G-site, GSH-binding site; H-site, hydrophobic-binding site; iprodione, ([3-(3,5-dichlorophenyl)-N-(1-methylethyl)-2,4-dioxo-1-imidazolidinecarboxamide]); Nb-GSH, S-(p-nitrobenzyl)glutathione.
