# Supplementary material for: Inhibition Analysis and High-Resolution Crystal Structure of Mus musculus Glutathione Transferase P1-1
Source: Biomolecules. 2023 Mar 29;13(4):613. doi: 10.3390/biom13040613 (PMC10136361; doi:10.3390/biom13040613)
Supplement: Supplementary file 1 [file biomolecules-13-00613-s001.zip › biomolecules-2282282-supplementary.pdf]

Supplementary Materials

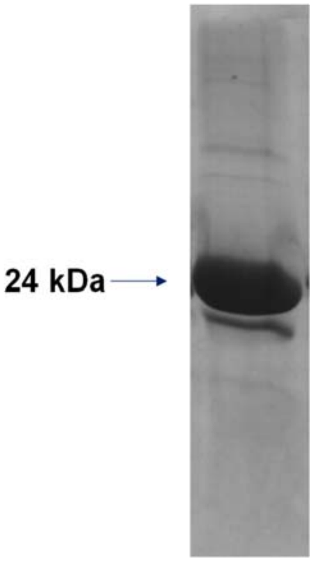

Figure S1. SDS-PAGE analysis of the eluted fraction of recombinant *Mm*GSTP1-1. Protein bands were stained with Coomassie Brilliant Blue R-250. *Mm*GSTP1-1 was purified by affinity chromatography using GSH-Sepharose as adsorbent.

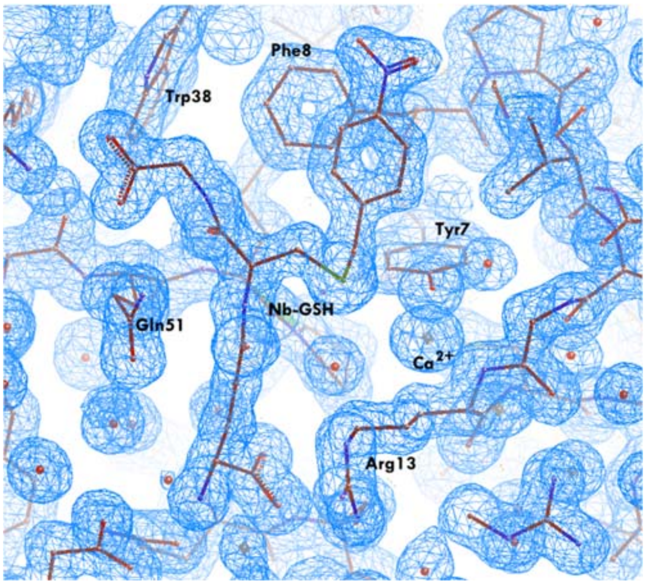

Figure S2. SigmaA-weighted 2Fo-Fc electron density map of Nb-GSH with surrounding residues at 1 sigma level. The figure was created using Coot [1].

References

[1]. Emsley, P.; Lohkamp, B.; Scott, W.G.; Cowtan, K. Features and development of Coot. Acta Crystallogr D Biol Crystallogr. 2010, 66, 486–501. doi: 10.1107/S0907444910007493. Epub 2010 Mar 24. PMID: 20383002; PMCID: PMC2852313.
